# Analysis of complete mitochondrial genome sequence of Kessleri thrush, *Turdus kessleri* (Passeriformes, Turdidae)

**DOI:** 10.1080/23802359.2018.1467222

**Published:** 2018-08-13

**Authors:** Sen Song, Jiahui Qin, Juanjuan Luo, Donghai Li, Bo Jiang, Cheng Chang

**Affiliations:** School of Life Sciences, Lanzhou University, Lanzhou, China

**Keywords:** *Turdus kessleri*, mitochondrial genome, conversation

## Abstract

In this study, the complete mitochondrial genome of *T. kessleri* was sequenced and characterized. The overall base composition of *T. kessleri* mitogenome is 29.3% for A, 32.3% for C, 15.0% for G, and 23.5% for T. The percentage of G + C content is 47.2%. The mitogenome is a circular DNA molecule of 16,754 bp in length, including a D-loop region, two rRNA genes (12SrRNA and 16SrRNA), 13 protein-coding genes (PCGs), and 22 tRNA genes. The sequence information of *T. kessleri* can contribute to enrich the molecular data resources about birds and could also enable the phylogenetic research and help to resolve phylogenetic relationship problems related to *Turdus*.

The Kessleri thrush, *Turdus kessleri*, belonging to Passeriformes, Turdidae, *Turdus*, was mainly distributed in China (Mackinnon et al. 2000; Del Hoyo et al. 2016; Zheng 2017). There are only a few researches on such bird, most of them are about its distribution (Mackinnon et al. 2000; Zheng 2017), breeding (Yang et al. 2012), and appearance, less is at the molecular level. Analysing the total mitochondrial genome (mtDNA) of *T. kessleri* can facilitate the studies on species origin, evolution, and phylogenesis, and may contribute to its conservation.

The complete mtDNA was extracted from blood tissue obtained from the samples collected in Luqu County (102°28′59.81″E, 34°39′50.73″N, altitude 1884 m), Gannan Tibetan Autonomous Prefecture, China and stored in the Molecular Ecology Lab of School of Life Sciences, Lanzhou University with the TIANamp Genomic DNA Kit (Tiangen, Beijing, China). 12S, 16S, ND2, ND4 and Cytb were amplified by universal primers (Kocher et al. 1989; Arevalo et al. 1994; Rassmann 1997). For long-and-accurate PCR (LA-PCR), eight pairs of specific primers were designed subsequently referred to those DNA sequences. PCR products were then sent to sequencing company directly to sequence, as some of the fragments are longer than 1 kb, primer walking was used.

The complete mitochondrial genome sequence of *T. kessleri* is a closed circular structure as the mtDNA of other birds. And for *T. kessleri*, it was 16,754 in length totally (GenBank No. MG912943) and composed of a D-loop region, two rRNA genes, 13 protein-coding genes (PCGs) and 22 tRNA genes. Among those genes, ND6, tRNA^Glu^, tRNA^Pro^, tRNA^Tyr^, tRNA^Cys^, tRNA^Asn^, tRNA^Ala^, and tRNA^Gln^ were all encoded on the light strand, while others were encoded on the heavy strand. ND2 and COI genes used GTG as their start codon and all other PCGs used ATG. Besides, all the PCGs were ended by codon TAA, AGA, TAG, AGG except for ND4 and COX3 that were ended by an incomplete stop codon, the single T. The base composition of the complete genome was about 29.3% for A, 23.5% for T, 32.3% for C, and 15.0% for G. The percentage of A + T content is about 52.8%, whereas the G + C content is about 47.3%. The length of D-loop, 12SrRNA, 16SrRNA, tRNA, and PCGs was 1199 bp, 985 bp, 1601 bp, 1547 bp, and 11,422 bp separately. There were 28 overlaps or gaps identified among those genes, the length of the overlaps and gaps were 44 bp and 69 bp, respectively.

Phylogenetic analysis of this study included mtDNA of seven species of the genus *Turdus*. The Bayesian tree showed that *T. kessleri* was clustered with *T. eunomus* with a high bootstrap value of 100 (Figure 1). Considering *T. kessleri* had been barely researched on the molecular level, the phylogenetic relationships could be taken as a reference for the whole genus *Turdus*. The sequence information of *T. kessleri* can contribute to enrich the molecular data resources about birds and could also enable the phylogenetic research and help to resolve phylogenetic relationship problems related to *Turdus*. Hopefully, our work may be useful for the conservation of the species in the future.

**Figure 1.**
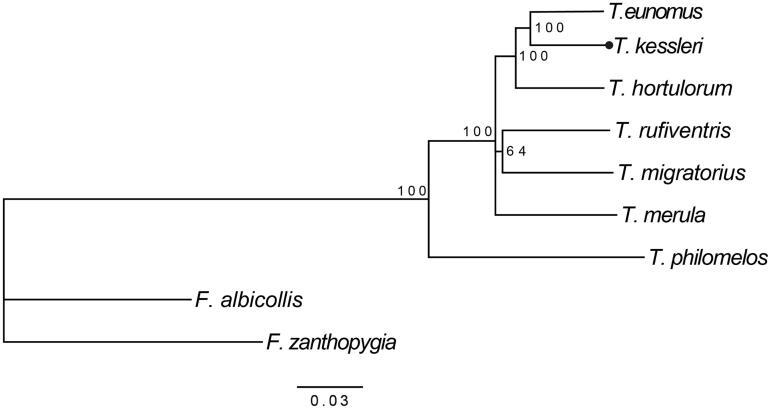
Bayesian tree of *T. kessleri* and other eight species among which *F. zanthopygia* and *F. albicollis* were used as an outgroup. Those numbers on nodes show Bayesian support values. Accession numbers of these nine species are listed as below: *T. kessleri* MG912943, *T. eunomus* KM015261, *T. hortulorum* KF926987, *T. rufiventris* KT346357, *T. migratorius* KJ909198, *T. merula* KT373849, *F. albicollis* KF293721, and *F. zanthopygia* JN018411.
